# A comparison between type 3 excision of the transformation zone by straight wire excision of the transformation zone (SWETZ) and large loop excision of the transformation zone (LLETZ): a randomized study

**DOI:** 10.1186/s12905-015-0174-5

**Published:** 2015-02-18

**Authors:** Fábio Russomano, Maria Aparecida Pereira Tristao, Renata Côrtes, Maria José de Camargo

**Affiliations:** Department of Gynecology, Colposcopy Clinic, Instituto Nacional de Saúde da Mulher, da Criança e do Adolescente Fernandes Figueira of Fundação Oswaldo Cruz (IFF/Fiocruz), Av. Rui Barbosa, 716, Flamengo, Rio de Janeiro, Brazil, CEP 22250-020; Department of Pathological Anatomy, IFF/Fiocruz, Rio de Janeiro, Brazil; Post-graduate Student at IFF/Fiocruz, Rio de Janeiro, Brazil

**Keywords:** Cervical intraepithelial neoplasia, Colposcopy, Diathermy, Treatment outcome, Uterine cervical neoplasm, Conization

## Abstract

**Background:**

The management of preinvasive cervical lesions has the objective to ensure the absence of invasive lesions and to prevent progression to cancer. Excisional procedures have been preferred to treat these lesions as they report the presence of unsuspected invasive lesions and the status of surgical margins, allowing inferring full excision when such are free of disease. The purpose of this study is to determine whether Straight Wire Excision of the Transformation Zone (SWETZ) is a better alternative than Large Loop Excision of the Transformation Zone (LLETZ-cone) as a type 3 excision of the Transformation Zone (TZ) to reduce incomplete excision and concerning other outcomes of surgical interest.

**Method:**

Randomized controlled trial including women who needed type 3 excision of the TZ referred to a colposcopy clinic after cytological screening between January 2008 thru December 2011. The interventions were performed using local anesthesia and sedation in an inpatient basis by different experienced surgeons. The study enrolled and randomized 164 women, of which 82 were allocated to each group. After exclusions, 78 remained in SWETZ and 76 in LLETZ-cone groups for the analysis of outcomes of surgical interest and 52 and 54, respectively, for the margins analysis.

**Results:**

There was an even distribution between the groups after randomization and exclusions, concerning mean age, parity, current smoking, prior cytological diagnosis and histopathological diagnosis obtained in cone specimen even after exclusions. We observed significantly higher risk of compromised or damaged endocervical margin in specimens resulting from the LLETZ-cone in relation to SWETZ (RR 1.72, 95% CI: 1.14 to 2.6), with an absolute risk reduction (ARR) of 26.4% (95% CI: 8.1 to 44.8) for patients operated by SWETZ. The specimens obtained by SWETZ showed less fragmentation (ARR = 19.8%, 95% CI: 10.3 - 29.3%), but the procedure took longer. There were complications in 5.6% of the procedures, with no significant differences between the groups.

**Conclusion:**

This study showed a lower proportion of compromised or damaged endocervical surgical margin in specimens resulting from SWETZ in relation to LLETZ-cone. SWETZ demonstrated to be more efficient than LLETZ-cone concerning less fragmentation of the specimen obtained. However, it accounted for longer surgical time. Both techniques showed morbidity

**Trial registration:**

Number at ClinicalTrials.gov: NCT01929993 (June 10, 2012).

## Background

Cervical cancer is still a leading cause of cancer-related deaths in developing countries. In Brazil, the official estimates for 2014 show incidence of 15.33 new cases of cervical cancer per 100,000 women [1].

The treatment of patients identified to have squamous cancer precursor lesions (cervical intraepithelial neoplasia grade 2 or 3 - CIN 2–3) may be excisional or destructive [2]. The excisional treatment has been preferred as it is as effective as the other methods and enables the diagnosis of invasive lesions and points out resection margins [2], which is an important prognostic factor for residual or recurrent lesions [3,4].

When the transformation zone (TZ) is located in the endocervical canal (type 3 TZ) or may involve cervical glandular epithelium, the recommended procedure is the conization (or type 3 excision of the TZ) [2]. However, excisional procedures have the potential for complications. The most common are per- or post-operatory bleeding [5], but the most important are perinatal morbidity, usually associated to more extensive removals of the endocervix [6] and cervical stenosis, which can impair follow-up [5]. This risk had led some authors to recommend less invasive procedures, as did Prendiville with Large Loop Excision of the Transformation Zone (LLETZ) [7]. This procedure may be applied in any circumstance to treat the ecto or endocervical TZ. However, it has been misused when a 10 mm deep loop electrode is used to remove lesions that extends into the endocervical canal (types 2 or 3 TZ). In such cases, there is a greater possibility of incomplete excision [2,8].

With the purpose to suggest the most appropriate treatment for these situations, Prendiville later proposed the Straight Wire Excision of the Transformation Zone (SWETZ), which is able to remove the TZ located in the endocervix with a possible lower morbidity, lower chance of incomplete removal and of segmentation of the specimen or excessive removal [9]. This procedure has also been referred in literature as “an excision using a fine needle” [10] or NETZ (*needle excision of the transformation zone*) [11-13].

A randomized study comparing NETZ and LLETZ showed a higher probability of disease free margins and lesser fragmentation of the specimens obtained by NETZ, but showed worse results concerning bleeding during the surgical procedure [13]. However, this study also included women with ectocervical disease and there were no published studies comparing the excisional electrosurgical techniques, such as LLETZ and SWETZ, used as type 3 excision, to excise endocervical disease.

A preliminary study of our group found SWETZ to be more efficient than LLETZ in endocervical disease, concerning compromised margins, but with no statistical significance [14].

The purpose of this study is to confirm if SWETZ is a better alternative than LLETZ (used as a type 3 excision of the TZ) concerning the reduction of incomplete excision (compromised surgical margins) of type 2 or 3 TZ and the morbidity related to the surgery and other variables of clinical interest: procedure related bleeding, fragmentation of the specimen and procedure time. Moreover, the differences between the ambition of excision by the surgeon and the specimen obtained were measured and compared in both techniques. To differentiate LLETZ as a type 3 excision of the TZ from the one used for ectocervical disease (type 1 excision of the TZ), this technique will be referred here as LLETZ-cone.

## Method

### Patient population

This was a randomized controlled trial comparing two techniques of electrosurgical type 3 excision of the TZ in a representative sample of women living in the city of Rio de Janeiro. Women were identified by cytological screening as possible carriers of invasive or preinvasive lesions and referred to the Colposcopy Clinic at Instituto Nacional de Saúde da Mulher, da Criança e do Adolescente Fernandes Figueira of Fundação Oswaldo Cruz (Fernandes Figueira National Institute for Women, Children, and Adolescent’s Health, Oswaldo Cruz Foundation) (IFF/Fiocruz), in Rio de Janeiro, Brazil. Eligible subjects must have one of the following indications for conization: suspicion of microinvasive carcinoma or cytological suspicion of cancer without visible lesion; cytological suspicion or for treatment of preinvasive glandular disease; type 2 or 3 TZ in patients with high grade intraepithelial lesions (CIN 2–3) confirmed by biopsy or diagnosed by Pap smear.

The study included the eligible women who agreed to participate and signed an informed consent. Women with history of previous treatment for preinvasive cervical lesions or, after being included, lost type 3 excision indication after randomization (ECJ turned fully visible during colposcopy just before procedure in the operating room), had colposcopically suspicion of invasion detected after inclusion, in which it was impossible to perform conization (due to important atrophy) or changed to the other arm of the study due to technical reasons were excluded from the surgical outcomes analysis. Other ones were excluded from margin analysis due to processing artifacts, absence of CIN2/3/IA1 carcinoma or presence of invasive disease on histopathological analysis.

### Sample size

To calculate the sample size, the study considered data from the preliminary study, mentioned above, which showed compromised margin in 4% of the specimens resulting from SWETZ, and in 20% from LLETZ-cone [14]. Thus, assuming the compromised margin risk hypothesis to be five times higher in LLETZ-cone than in SWETZ, a 5% alpha error and Power of 80%, we calculated the need to include 152 patients, 76 for each group (Epi-INFO v. 6.04). Considering the possibility of up to 10% of losses or exclusions after randomization, we recruited a total of 168 patients.

### Randomization

We applied a block randomization to produce an equal number of patients in each group [15]. The resulting allocation list was used to organize opaque sealed envelopes containing one of the possible allocations. They were externally numbered in sequence and given to the surgeon only after the inclusion of each patient in the study, by a collaborator (in the operating room). Thus, the surgeon was aware of the patient's allocation only when she was included and minutes before performing the procedure.

### Surgical technical methods

The procedures were performed through local anesthesia and sedation in an inpatient basis by several surgeons with different expertise. LLETZ-cone was performed with a 20 mm high loop electrode, in which the activated electrode is laterally applied to the margin of the cervical TZ and slid slowly up to the contralateral margin of the TZ, with the purpose to remove 20 mm depth of the endocervical epithelium. After specimen removal, the crater was fully fulgurated with a ball electrode. The surgeons used standard Valleylab Force 2 electrosurgical units or similar, and blend 2 wave, in which there is 50% of cut and 50% of coagulation, with 50-50 W of power range, as recommended by Prendiville [2].

SWETZ uses a 10 mm length straight electrode and 0.2 mm of straight section, applied similarly to a bistoury or laser, shaping the cone according to the desired dimensions. After circular incision in the ectocervical area, the incision progresses toward the cervical stroma, forming an angle of approximately 45 degrees with the surface and moving into the cervical canal. As in LLETZ, the crater was fully fulgurated with a ball electrode. The cone endocervical extension is defined based on studies that show that the endocervical disease is located in the lower third of the cervical canal, even when glandular [16]. This technique is similar to cold knife conization, however, it uses diathermy for cutting and coagulation of the surgical wound. To minimize thermal damage and ensure proper orientation in the removal and examination of the surgical specimen, the cone must be removed in one single piece. This procedure used, as standard in the electrosurgical units, blend 2 wave for the initial incision and plain coagulation in the stroma, or blend 3 throughout the procedure, both options with 50-50 W of power.

### Outcome measures

Blood loss was visually estimated by the surgeon during the procedure and categorized as less than 20 ml (or insignificant), between 20–100 ml (moderate), and above 100 ml (significant). Complications, when present, were described. The number of fragments that composed the surgical specimen was recorded by the surgeon. The procedure time was measured by nurses in the operating room from the insertion up to the removal of the vaginal speculum, registering the beginning and the end of the procedure. The ambition of the excision was estimated by the surgeon based on the extension of the ectocervical TZ associated with the endocervical length. This measure was verified by cervicometry, prior to the intervention, using a hysterometer. Based on this assessment, the surgeon would choose the 15 or 20 mm width loop, depending on the ectocervical extension of the TZ, but both with 20 mm of height, for patients allocated to LLETZ-cone, in order to excise this extension of the endocervical canal, or would define the cone base circumference and its height, for those allocated to SWETZ. The sum of these measurements were recorded as geometric ambition of excision and later compared with the dimensions of the specimen fixed in formaldehyde, measured by a single pathologist with extensive experience in the examination of this material (MAPT).

After excision, one of the margins was marked with a pin to guide the pathologist and the surgical specimen was opened and fixed on a styrofoam plate before immersion in formaldehyde 10% [2]. The specimens obtained were sent for histopathological examination without the information of which surgical technique was used. After fixation, the specimens had their margins painted in nankin in different colors, according to the surgeon's advice in order to let it be correctly identified during histopathological analysis.

The study considered as compromised margin the one in which CIN 2–3 or microinvasive neoplasia reached the limit of the excised specimen. The impaired margin was the one with any alteration that would harm its evaluation in at least one fragment or histological cut (e.g. those detaching because of thermal damage or segmentation). The thermal artifact resulting from the electrosurgical excision, was measured in percentage of cuts hindering the histological assessment of the margins or histological diagnosis of the specimen. All specimens were examined by the same experienced pathologist (MAPT).

### Data collection and statistical analysis

Data were stored in Microsoft Access 2000 database and analyzed using EpiInfo v.7 and SPSS v.21. The means, medians and proportions were compared, the Absolute Risk Reduction (ARR), Relative Risk (RR) and Number Needed to Treat (NNT) were calculated with confidence intervals of 95% (95% CI) and the Student *t*-test, Mann–Whitney, Chi-square and Fisher's Exact tests were applied for statistic significance where appropriated. The study considered significant values of p < 0.05. The Power of the study to the blood loss analysis was calculated using DSS Research Statistical Power Calculators [17].

### Follow-up

Data concerning residual or recurrent disease and late complications like stenosis will not be described in this report because patients with disease free margins are followed up in basic care health units. A new study is going on to address these outcomes in a future publication.

This study was approved by the Committee for Ethics in Research involving human beings from IFF/Fiocruz and registered at ClinicalTrials.gov under # NCT01929993.

## Results

**Figure 1:**
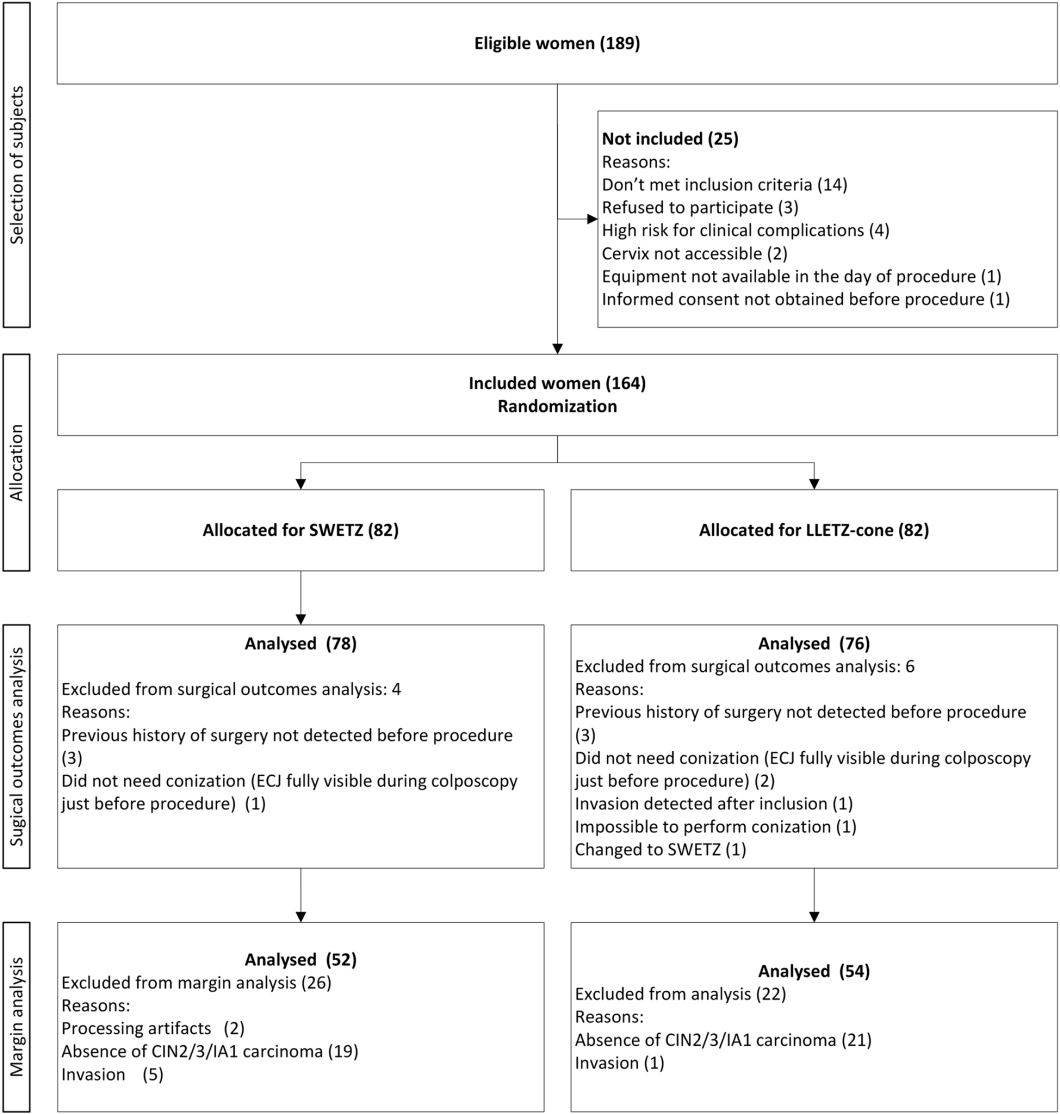
**Flow chart of the study.**

Out of the 189 eligible women, 164 were included after signing an informed consent, between January, 2008 thru December, 2011, and randomized to each group. Eighty-two women were allocated for SWETZ and 82 for LLETZ-cone (Figure 1). The cases we noticed that met an exclusion criteria after allocation or did not received the procedure to which were allocated to, were excluded from the analysis. Moreover, cases that did not confirm microinvasive or preinvasive disease or showed invasive disease of the cervix in the histopathological analysis were excluded from the margin analysis because the access of surgical margins in these cases are not useful.

After these exclusions, 156 remained, 78 for SWETZ, and 76 for LLETZ-cone for the analysis of surgical outcomes, and 106 patients remained for the margins analysis (52 allocated for SWETZ and 54 for LLETZ-cone) (Figure 1).

There was an even distribution between the groups after randomization, even after the exclusions, concerning mean age, parity, current smoking, cytopathological diagnosis prior to conization and histopathological diagnosis obtained in the conization specimen. Most women were in the fifth decade of life, with average parity of three children and cytological diagnosis of high grade intraepithelial lesion (HSIL). In the SWETZ group, there were five cases with cytological suspicion of microinvasion (6.4%), but histopathological analysis didn’t confirm such difference. The main histopathological diagnosis in both groups was preinvasive disease, mostly CIN 3. No significant difference was observed in the cytological diagnosis and final histopathology of the cone or regarding the cervical canal size. In almost 90% of the cases, in both groups, the reason for indicating the cone was type 2 or 3 TZ (Table 1). The absence of statistical significant differences was observed also after exclusions from the margin analysis (data not shown).

**Table 1:** **Comparison between the groups (SWETZ × LLETZ-cone): characteristics of patients (IFF/Fiocruz - 2008–2011)**

**Variable**	**SWETZ**	**% or SD**	**LLETZ-cone**	**% or SD**	**p-value**
N	78	50,6	76	49,4	-
Mean age	45.07	11.54	43.25	10.59	0.3
Mean parity	2.93	1.93	3.04	2.27	0.7
Smoking	25	32.1	27	35.5	0.6^†^
# of cigarettes/day in smokers	18.95	10.41	16.08	6.11	0.3*
Cytological diagnosis (n)					
AdenoCA	1	1.3	1	1.3	
AGC (NOS)			1	1.3	
AGC-H	1	1.3	2	2.6	
AGC-US	1	1.3		0.0	
AIS	5	6.6	2	2.6	
ASC-H	7	9.2	11	14.1	
ASC-US	2	2.6		0.0	
Cancer	3	3.9	3	3.8	
HSIL	54	71.1	51	65.4	
HSIL (possible microinvasion)		0.0	5	6.4	
LSIL	1	1.3	2	2.6	
Negative	1	1.3		0.0	-
Indication of conization					
AIS cytology	6	7.9	6	7.7	
Cancer cytology	1	1.3	1	1.3	
Type 2 TZ^||^	1	1.3	1	1.3	
Type 3 TZ	68	89.5	70	89.7	0.9^†‡^
Histopathological diagnosis of the cone (n)					
Inconc/impaired	3	3.84	-	0	
Negative	8	10.26	9	11.84	
CIN 1	9	11.54	12	15.79	
CIN 2/3	51	65.38	52	68.42	
Cancer	7	8.97	3	3.95	0.5^†**^ 0.3^§††^
Cervicometry (mm)	27.84	7.74	27.95	7.71	0.9^*^

^*^Student’s *t*-test.

^†^Chi-square.

^‡^Obtained from recategorization in type 3 TZ versus other categories.

^§^Fisher's Exact Test.

^||^Transformation Zone.

^**^Obtained from recategorization in preinvasive or invasive disease versus others.

^††^Obtained from recategorization in preinvasive versus invasive disease.

The surgical outcomes observed in each group are shown in Table 2. Fragmentation (more than one piece) of the surgical specimen and the surgery time showed statistically significant difference between the groups. Fragmentation during excision was significantly higher in the LLETZ-cone group. Patients undergoing SWETZ had ARR of fragmentation of the specimen removed of 19.8% (95% CI: 10.3- 29.3%) compared to LLETZ-cone.

**Table 2:** **Comparison between the groups (SWETZ × LLETZ-cone): surgical outcomes (IFF/Fiocruz - 2008–2011)**

	**SWETZ**	**%, range or SD** ^*^	**LLETZ-cone**	**%, range or SD** ^*^	**ARR (95% CI)**	**RR (95% CI)**	**p-value**
N	78	100	76	100	-	-	-
Blood loss (ml)							
<20	74	94.9	76	100	-	-	
20-100	4	5.1	0	0	−5.1 (−10.0 – -0.2)	-	0.1^†^
Number of segments removed							
1	77	100	59	78.7	19.8 (10.3-29.3)	-	-
>1	0	0	16	21.3	-	-	<0,01^‡^
No data	1	-	1	-		-	-
Surgery time - minutes (median)	20	8-60	17	9-32	-	-	<0,01^§^
≤18 min	33	42.9	44	60.3	-	-	-
>18 min	44	57.1	29	39.7	−16.3 (−32,2 – -0.4)	1.44 (1.02-2.03)	
No data	1	-	5	-	-	-	-
Geometric ambition - mm (average)^¶^	30.92	6.31	30.54	6.15	-	-	0,7^||^
Geometric ambition difference - mm (average)^¶**^	10.19	5.7	10.62	5.5	-	-	0,7^||^
Anesthetic volume – ml	8.26	1.95	8.04	1.8	-	-	0,5^||^
No data	11	-	15	-	-	-	-
Complications	2^‡‡^	2.6	2^††^	3	0.1 (−5.0-5.1)	0.97 (0.14-6.74)	1^†^

^*^Cases with no data were excluded.

^†^ Fisher's Exact test.

^‡^Chi-square.

^§^Mann–Whitney test.

^||^Student's *t*-test.

^¶^The ones unfilled of ambition or without histological measure were excluded.

^**^Difference between the desired extension (endo + ecto) and what was effectively measured in the specimen.

^††^Opening of the vaginal mucosa and the need for anesthetic supplementation.

^‡‡^Opening of the lateral vaginal cul-de-sac with vascular injury, requiring total hysterectomy and need for general anesthesia.

However, the length of the procedure was longer in the group undergoing SWETZ. Taking into account the surgery median time (18 min), it was possible to calculate the risk of surgical time above this limit. Women undergoing SWETZ had RR of 1.44 (95% CI: 1.02 - 2.03) to have surgery time above 18 min than the group that underwent LLETZ-cone. Blood loss was less than 20 ml in most procedures in both groups, one out of every four women having bleeding between 20–100 ml, all in SWETZ group (ARR = 5.1%, 95% CI: − 0.2 to 10.0).

The geometric ambition of epithelial excision, the difference between this measure and the one of the specimen effectively removed and the volume of local anesthetic used did not differ between the groups. Regarding geometric ambition, in both techniques, the surgeon planned a full excision of tissue of approximately 10 mm larger than what was actually measured in the surgical specimen, during the histopathological analysis.

Complications occurred in 5.6% of the procedures: one woman in the LLETZ-cone group had an accidental opening of the vaginal mucosa up to near the muscle layer of the bladder that needed to be repaired and another woman needed general anesthesia due to some discomfort during the procedure. In the SWETZ group, there was an accidental opening of the lateral vaginal cul-de-sac with vascular injury, requiring total hysterectomy in a case and another one needed general anesthesia, also due to some discomfort during the procedure (2.6%).

Table 3 shows the results related to the surgical compromised margin in each one of the groups. There was a significantly higher risk of compromised or impaired endocervical margin in the conization specimen resulting from LLETZ-cone compared to SWETZ (RR = 1.72, 95% CI: 1.14-2.6), with an ARR of 26.4% (95% CI: 8.1-44.8) for patients who underwent SWETZ. For each 4 patients who underwent SWETZ, one less showed endocervical compromised or impaired margin compared to those who underwent LLETZ-cone (NNT = 4, 95% CI: 2.2-12.4).

**Table 3:** **Comparison between the groups (SWETZ x LLETZ-cone): analysis of surgical margins in cone specimen by procedure (IFF/Fiocruz - 2008–2011)**

**Procedure outcome**	**SWETZ**	**%**	**LLETZ-cone**	**%**	**p-value**	**RR** ^*^ **(95% CI)**
Ectocervical margin						
Free	37	71.2	32	59.3		
Compromised	4	7.7	9	16.7		
Impaired	11	21.2	13	24.1	0.2^*^	1.41 (0.83-2.41)
Totals	52	100	54	100		
Endocervical margin						
Free	33	63.5	20	37.0		
Compromised	7	13.5	15	27.8		
Impaired	12	23.1	19	35.2	<0.01^†^	1.72 (1.14-2.6)^‡^
Totals	52		54			
Stromal margin						
Free	50	96.2	50	92.6		
Compromised	2	3.8	2	3.7		
Not evaluated		0.0	1	1.9		
Impaired		0.0	1	1.9	0.6^§^	1.47 (0.26-8.45)
Totals	52	100	54	100		
Some margin^||^						
All free	24	46.2	14	26.4	0.03^†^	1.37 (1.01-1.84)^‡^
Some margin compromised or impaired	28	53.8	39	73.6		
Totals	52		53			
None impaired	32	61.5	29	54.7	0.5^†^	1.18 (0.75-1.85)
Some impaired	20	38.5	24	45.3		
Totals	52		53			

^*^For this calculation, the histopathological diagnosis of margin involvement and impaired were grouped. The undesired outcome was "margin involvement or impaired" and the risk factor "LLETZ".

^†^Chi-square test for analysis of "margin involvement or impaired” *vs*. “free” by procedure made.

^‡^RAR = 26.4% (95% CI, 8.1-44,8) and NNT = 3 (95% CI, 2.2-12.4).

^§^Fisher's exact test for analysis of "margin involvement or impaired" by procedure, excluding the "not evaluated" one.

^||^Excluding the case of "not assessed" stromal margin.

^‡^RAR = 19.7% (95% CI, 1.7-37,8) and NNT = 5 (95% CI, 2.6-58.0).

^**^Chi-square test.

Similar benefits were observed in the analysis of any compromised or impaired margin in the surgical specimen: RR was 1.37 (95% CI: 1.01-1.84) for this finding in conization specimen obtained by LLETZ-cone in relation to SWETZ, with ARR of 19.7% (95% CI: 1.7-37.8) in favor of SWETZ and NNT of 5 (95% CI: 2.6-58.0). No significant differences were observed between the groups concerning other margin involvement or impairment.

Concerning the reasons that hindered the surgical margins analysis, we hadn’t found any statistically significant differences between groups (data not shown). Despite the high percentage of thermal artifact hindering the margin analysis, the proportion of uninterpretable margins or of loss of the histopathological diagnosis, partial or total, was much lower and, once again, there was no statistical difference between the groups. The average proportion of cuts with partially impaired diagnosis was 6.5% (in 5 cases) in the LLETZ-cone group and 6.6% (in 2 cases) in the SWETZ arm, and, considering the ones that totally impaired diagnosis, these numbers were 3.8% and 1.5%, in the same cases, respectively (data not shown).

## Discussion

In this study, SWETZ presented significant lesser risk of compromised surgical margins or specimen fragmentation, but took longer time than LLETZ-cone to be performed.

Two meta-analysis demonstrate the importance of margin involvement as an indicator of incomplete treatment [3,4], which has led some researchers to seek excisional techniques to reduce the limitations of LLETZ [2,13]. In the most conservative assumption, the risk of endocervical compromised or impaired margin was, in our study, 14% higher in LLETZ-cone than in SWETZ, and one in every four patients that underwent SWETZ did not have, in comparison to the ones that underwent LLETZ-cone, this undesired outcome. Concerning the ectocervical or stromal margins, there was a greater number of compromised or impaired margins in the LLETZ-cone group than in SWETZ, however with no statistical significance. The superiority of SWETZ over LLETZ-cone could also be observed when analyzing the compromised or impaired diagnosis in any of the surgical margins (endocervical, ectocervical or stromal): the specimens obtained by SWETZ had about 20% less compromised or impaired margins, when analyzed together, than LLETZ-cone. Although impaired margin is not the same as compromised one, clinically it has the same significance to post-treatment follow-up. The high percentage of impaired margins reflects a feature of specimens resulting from electrosurgical excisional procedures, systematically reported by our pathologists, but, as said above, this fact did not interfere with final diagnosis.

There were no cases with unknown margin or even of evaluation hindered by fragmentation of the specimen, possibly due to the care taken in preparing the specimen and the histological cuts, as described above. Previous experimental studies addressing the same question showed no statistically significant differences between the two groups [13,14]. This may be explained by lack of Power due to the small sample size of the mentioned studies and to the presence of some cases with unknown margin, which was not observed in our study.

The use of the straight electrode significantly reduced the possibility of fragmentation, which allows better study of the margins and an adequate diagnosis of early invasion. This result is consistent with what was observed in other two studies mentioned previously [13,14].

The technique of straight electrode takes longer than using a loop electrode as it needs the cone drawing by the surgeon. The loop conization was performed in less time and this may represent a benefit to patients that need a shorter surgical procedure. Although this difference, surgical time in the two procedures was higher than in other studies [13,14]. However, in the present trial, it was considered since the insertion of the vaginal speculum until its removal, which includes a colposcopic reevaluation, anesthetic infiltration and cervicometry, instead of considering it from the surgical incision to specimen removal, as in the other ones.

Concerning blood loss, the amount of bleeding in each arm was not statistically different, although Panoskaltisis *et al*. found more bleeding for SWETZ [13]. This lack of significant difference in bleeding can be attributed to surgeons more familiar with the technique, in addition to the choices on the type of cutting wave and of coagulation used. Another possibility is the lack of Power to detect this difference. In this analysis, the Power was calculated as 61.8%.

Despite the small number of patients, both techniques showed morbidity and the most significant complication was the need for hysterectomy in only one patient in the SWETZ arm, as a consequence of a less experienced surgeon performing the procedure.

The difference observed regarding the geometric ambition and the specimen size actually removed is partially explained by the retraction the specimen undergoes during fixation with formaldehyde [18], but also by the difficulty of the surgeon to meet the cone geometric planning.

A question that remains is if the two procedures remove similar amount of tissue, why one of them offers less compromised margins? Probably, other factors are responsible for fewer diagnoses of compromised margins in the SWETZ arm. In the present study, fragmentation and thermal artifact may have favor SWETZ. However given the small numbers, it was not possible to adjust the risk of compromised or impaired margins to these factors.

This trial has other limitations. The existence of cases excluded by history of previous surgery undetected prior to the inclusion of the participants and loss of the conization indication, accounted for few exclusions. After the allocation, in case the study concluded that the patient should not have been included, with the purpose to preserve the randomization, we chose to keep the inclusion and exclude it from the analysis. Patients who could not undergo surgery by a technique were also excluded, and underwent surgery by the competing technique. The exclusions did not result in imbalance of the groups concerning sociodemographic and clinical characteristics.

Several exclusions took place in both groups for the margin analysis and they were related to events not related to the procedures or to the outcomes. Most exclusions were due the absence of preinvasive or microinvasive disease in the conization specimen, in which case it would be impossible to count for the compromised margin outcome. Another reason for exclusion was the presence of invasive disease, a condition that increases the risk of compromised margin and this information loses clinical relevance, once the treatment will no longer be conservative. Blood loss and geometric ambition could be biased, because they were estimated by the surgeons, already knowing patient allocation, but the differences in these outcomes did not reach statistical significance. Finally, there was low Power to detect statistical differences in analysis of thermal artifact and cuts with impaired diagnosis due to thermal artifact, as happened with the blood loss analysis.

## Conclusion

This study demonstrates that SWETZ is more effective than LLETZ-cone concerning risk of compromised or impaired endocervical surgical margin. SWETZ also demonstrated superiority to LLETZ-cone regarding fragmentation of the obtained specimen. However, it accounted for longer surgical time. Both techniques showed morbidity. These features should be considered when choosing the surgical technique for women that need a type 3 excision for diagnosis and treatment of cervical preinvasive lesions.
